# First Insight into the Genotypic Diversity of Clinical *Mycobacterium tuberculosis* Isolates from Gansu Province, China

**DOI:** 10.1371/journal.pone.0099357

**Published:** 2014-06-09

**Authors:** Jie Liu, Chongxiang Tong, Jiao Liu, Yuan Jiang, Xiuqin Zhao, Yuanyuan Zhang, Haican Liu, Bing Lu, Kanglin Wan

**Affiliations:** 1 State Key Laboratory for Infectious Disease Prevention and Control, National Institute for Communicable Disease Control and Prevention, Chinese Center for Disease Control and Prevention, Changping, Beijing, People's Republic of China; 2 Collaborative Innovation Center for Diagnosis and Treatment of Infectious Diseases, Hangzhou, Zhejiang, People's Republic of China; 3 Lanzhou Pulmonary Hospital, Lanzhou, Gansu, People's Republic of China; 4 Beijing Chaoyang District Center for Disease Control and Prevention, Chaoyang, Beijing, People's Republic of China; Institut Pasteur de Lille, France

## Abstract

**Background:**

Investigations of *Mycobacterium tuberculosis* genetic diversity in China have indicated a significant regional distribution. The aim of this study was to characterize the genotypes of clinical *M. tuberculosis* isolates obtained from Gansu, which has a special geographic location in China.

**Methodology/Principal Findings:**

A total of 467 clinical *M. tuberculosis* strains isolated in Gansu Province were genotyped by 15-locus mycobacterial interspersed repetitive units–variable number tandem repeats (MIRU-VNTR) and spoligotyping. The results showed that 445 isolates belonged to six known spoligotype lineages, whereas 22 isolates were unknown. The Beijing genotype was the most prevalent (87.58%, n = 409), while the shared type 1 was the dominant genotype (80.94%, n = 378). The second most common lineage was the T lineage, with 25 isolates (5.35%), followed by the H lineage with 5 isolates (1.07%), the MANU family (0.64%, 3 isolates), the U family (0.43%, 2 isolates) and the CAS lineage with 1 isolate (0.21%). By using the VNTR15_China_ method, we observed 15 groups and 228 genotypes among the 467 isolates. We found no association between the five larger groups (including the Beijing genotype) and sex, age, or treatment status, and there was no noticeable difference in the group analysis in different areas. In the present study, seven of the 15 MIRU-VNTR loci were highly or moderately discriminative according to their Hunter-Gaston discriminatory index.

**Conclusions/Significance:**

The Beijing genotype is the predominant genotype in Gansu province. We confirm that VNTR15_China_ is suitable for typing Beijing strains in China and that it has a better discriminatory power than spoligotyping. Therefore, the use of both methods is the most suitable for genotyping analysis of *M. tuberculosis*.

## Introduction

Tuberculosis (TB) is a serious public health problem. Its causative agent, *Mycobacterium tuberculosis* (*M. tuberculosis*), may have killed more persons than any other microbial pathogen [1]. To date, TB remains a worldwide public health threat, with estimated 8.7 million new cases in 2011 and 1.4 million deaths [2]. In China, the average prevalence of TB is 99 per 100,000, the highest absolute number of cases annually worldwide [3].

Modern epidemiological studies have surveyed the infection outbreak at the molecular level and tracked the spread of given *M. tuberculosis* strains to discover its mechanism of transmission. Genotyping the strains isolated from TB patients plays a key role in detecting the source of infection [4]. IS*6110* restriction fragment length polymorphism (IS*6110*-RFLP) typing of *M. tuberculosis* has been the most widely applied and standardized molecular typing methods. However, this method is labor intensive and requires culturing large quantities of slow-growing *M. tuberculosis*. Moreover, when strains harbor no copies, a single copy, or a few copies of IS*6110* in their chromosomes, the discriminating efficiency of this method is lowered dramatically. In this regard, many polymerase chain reaction (PCR)-based methods were developed to avoid the technical demand of IS*6110*-RFLP, such as multiple locus variable number tandem repeat (VNTR) analysis (MLVA) and spacer oligonucleotide typing (spoligotyping) [5], [6].

**Figure 1:**
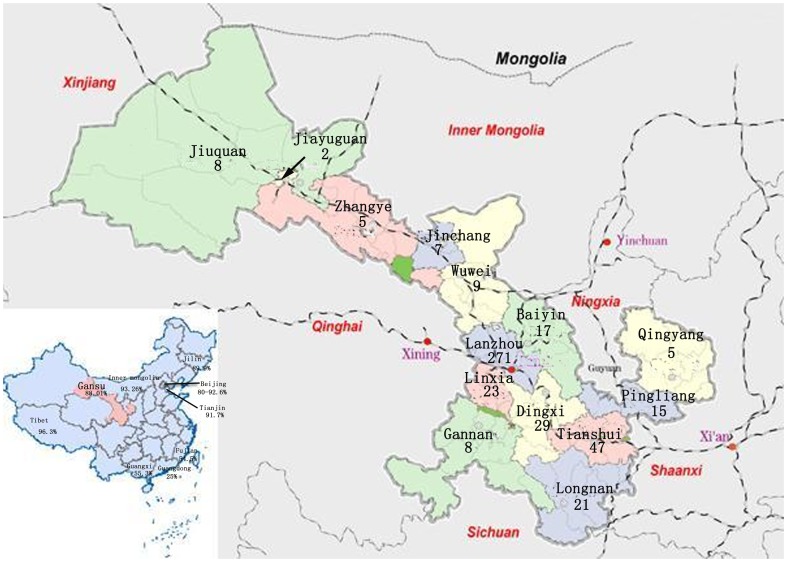
Map of Gansu Province and its location in China, and the proportion of the Beijing genotype isolates in several Provinces.

Gansu Province, located within the area of the Qinghai-Tibet, Loess, and Mongolian Plateaus in northwestern China and neighboring Mongolia (Figure 1), is a strip of land with a complicated geography and landform that separates the eastern and western sides of Northern China. It was the strategic passage and communications hub of Silk Road in ancient China, but is, now, one of the economically underdeveloped areas in China. Based on the 2010 national TB epidemiology survey in China, the prevalence rate of TB in Gansu is 298 per 100,000, higher than that of eastern China but lower than that of western China, posing a serious threat to the public health [7]. Hence, investigating the strain genetic diversity and the prevalence of Beijing genotype strains in Gansu is of utmost importance. We previously described the VNTR15_China_ typing scheme, and used it to analyse the genetic diversity of a large collection of isolates from more than 13 provinces in China [19], [33]. In the present study, we evaluated the discriminatory power of this scheme to characterize the strains from Gansu Province.

## Results

### Description of isolates

A total of 467 clinical *M. tuberculosis* isolates were collected from 2005 to 2011: 75 strains in 2005, 71 strains in 2006, 54 strains in 2007, and 267 strains in 2011. These clinical isolates were distributed throughout 14 regions in Gansu Province: 271 isolates from Lanzhou, 47 from Tianshui, 29 from Dingxi, 23 from Linxia Hui Autonomous Prefecture, 21 from Longnan, 17 from Baiyin, 15 from Pingliang, 9 from Wuwei, 8 from Gannan Tibetan Autonomous Prefecture, 8 from Jiuquan, 7 from Jinchang, 5 from Zhangye, 5 from Qingyang, and 2 from Jiayuguan (Figure 1). The patients with TB included 281 males and 186 females, with a median age of 41 (range from 7 to 85). A total of 269 patients had new-onset TB, while the remaining 198 patients were undergoing retreatment.

### Spoligotyping

We recognized a total of 44 distinct spoligotypes among the 467 isolates (Table 1). Typing analysis revealed that 441 isolates were grouped into 18 spoligotype clusters containing 2 to 378 isolates, while the other 26 isolates harbored unique spoligotypes.

**Table 1:** Spoligotypes of the *Mycobacterium tuberculosis* isolates (n = 467).

No.	Spoligotype	SIT^a^	Family^b^	N (%)^c^
1	□□□□□□□□□□□□□□□□□□□□□□□□□□□□□□□□□□▪▪▪▪▪▪▪▪▪	1	Beijing	378 (80.94)
2	□□□□□□□□□□□□□□□□□□□□□□□□□□□□□□□□□□▪▪▪▪▪□▪▪▪	190	Beijing	17 (3.64)
3	□□□□□□□□□□□□□□□□□□□□□□□□□□□□□□□□□□▪▪▪▪▪▪□▪▪	941	Beijing	1 (0.21)
4	□□□□□□□□□□□□□□□□□□□□□□□□□□□□□□□□□□▪□▪▪▪▪▪▪▪	621	Beijing	2 (0.43)
5	□□□□□□□□□□□□□□□□□□□□□□□□□□□□□□□□□□□□▪▪▪▪▪▪▪	269	Beijing	1 (0.21)
6	□□□□□□□□□□□□□□□□□□□□□□□□□□□□□□□□□□▪▪□▪▪▪▪▪▪	26	Beijing	3 (0.64)
7	□□□□□□□□□□□□□□□□□□□□□□□□□□□□□□□□□□▪▪▪▪□▪▪▪▪	255	Beijing	1 (0.21)
8	□□□□□□□□□□□□□□□□□□□□□□□□□□□□□□□□□□▪▪▪□▪▪▪▪▪	632	Beijing	1 (0.21)
9	□□□□□□□□□□□□□□□□□□□□□□□□□□□□□□□□□□▪▪▪▪□□□▪▪	NEW	Beijing	3 (0.64)
10	□□□□□□□□□□□□□□□□□□□□□□□□□□□□□□□□□□▪▪▪▪▪▪□□▪	NEW	Beijing	1 (0.21)
11	□□□□□□□□□□□□□□□□□□□□□□□□□□□□□□□□□□□□▪▪▪▪▪▪▪	NEW	Beijing-like	1 (0.21)
12	▪▪▪▪▪▪▪▪▪▪▪▪▪▪▪▪▪▪▪▪▪▪▪▪▪▪▪▪▪▪▪▪□□□□▪▪▪▪▪▪▪	53	T1	9 (1.93)
13	▪□▪▪▪▪▪▪▪▪▪▪▪▪▪▪▪▪▪▪▪▪▪▪▪▪▪▪▪▪▪▪□□□□▪▪▪▪▪▪▪	334	T1	4 (0.86)
14	▪▪▪▪▪▪▪▪▪▪▪▪▪▪▪▪▪▪▪▪□▪▪▪▪▪▪▪▪▪▪▪□□□□▪▪▪▪▪▪▪	291	T1	1 (0.21)
15	□▪▪▪▪▪▪▪▪▪▪▪▪▪▪▪▪▪▪▪▪▪▪▪▪▪▪▪▪▪▪▪□□□□▪▪▪▪▪▪▪	7	T1	2 (0.43)
16	▪▪▪▪□▪▪▪▪▪▪▪▪▪▪▪▪▪▪▪▪▪▪▪▪▪▪▪▪▪▪▪□□□□▪▪▪▪▪▪▪	154	T1	1 (0.21)
17	▪▪▪▪▪▪▪▪▪▪▪▪▪▪▪▪▪▪▪▪□□□□□▪▪▪▪▪▪▪□□□□▪▪▪▪▪▪▪	230	T1	1 (0.21)
18	▪▪▪▪▪▪▪▪▪▪▪▪▪▪▪▪▪▪▪▪▪▪▪▪▪▪▪▪□▪▪▪□□□□▪▪▪▪▪▪▪	462	T1	1 (0.21)
19	▪▪▪▪▪▪▪▪▪▪▪▪▪▪▪▪▪▪▪▪▪▪▪□□▪▪▪▪▪▪▪□□□□▪▪▪▪▪▪▪	1580	T1	1 (0.21)
20	▪▪▪▪▪▪▪▪▪▪▪▪▪▪▪▪▪▪▪▪□▪▪▪▪▪▪▪▪▪▪▪□□□□▪▪▪□▪▪▪	175	T2	2 (0.43)
21	▪▪▪▪▪▪▪▪▪▪▪▪▪▪▪▪▪▪▪▪▪▪▪▪▪▪▪▪▪▪▪▪□□□□▪▪▪□▪▪▪	52	T2	1 (0.21)
22	▪▪▪▪▪▪▪▪▪▪▪▪□▪▪▪▪▪▪▪▪▪▪▪▪▪▪▪▪▪▪▪□□□□▪▪▪▪▪▪▪	37	T3	2 (0.43)
23	▪□▪▪▪▪▪▪▪▪▪▪▪▪▪▪▪▪▪▪▪▪▪▪▪▪▪▪▪▪▪▪□□▪▪▪▪▪▪▪▪▪	1096	MANU2	3 (0.64)
24	▪▪▪□□□□▪▪▪▪▪▪▪▪▪▪▪▪▪▪▪□□□□□□□□□□□□□□□□▪▪▪▪▪	1789	CAS1-DELHI	1 (0.21)
25	▪□▪▪▪▪▪▪▪▪▪▪▪▪▪▪▪▪▪▪▪▪▪▪▪▪▪▪□□□▪□□□□▪▪▪▪▪▪▪	127	H4	4 (0.86)
26	▪▪▪▪▪▪▪▪▪▪▪▪□▪▪▪▪▪▪▪▪▪▪▪▪▪▪▪□□□▪□□□□▪▪▪▪▪▪▪	35	H4	1 (0.21)
27	▪▪▪▪▪▪▪▪▪▪▪▪▪▪▪▪▪▪▪▪▪▪▪▪▪▪▪▪▪▪▪▪□□□□□▪▪□▪▪▪	1098	U	2(0.43)
28	▪▪▪▪▪▪▪▪▪▪▪▪▪▪▪▪▪▪▪▪□▪▪▪▪▪▪▪▪□▪▪□□□□□□□▪▪▪▪	NEW	Unknown	1 (0.21)
29	▪▪▪▪▪▪▪▪▪▪□□□□□▪▪▪▪▪▪▪▪▪▪▪▪▪▪▪▪▪□□□□▪▪▪▪▪▪▪	NEW	Unknown	1 (0.21)
30	▪▪▪▪▪▪▪▪▪▪▪▪▪▪▪▪▪▪▪▪▪▪▪▪▪▪▪▪▪□▪▪□□□□□□□▪▪▪▪	NEW	Unknown	2 (0.43)
31	▪□▪□▪▪▪▪▪▪▪▪▪▪▪▪▪▪▪▪▪▪▪▪▪▪▪▪▪▪▪▪□□□□▪▪▪▪▪▪▪	NEW	Unknown	2 (0.43)
32	▪▪▪▪▪▪▪▪▪▪▪▪▪▪▪▪▪▪▪▪□□▪▪▪▪▪▪▪▪▪▪□□▪▪▪▪▪▪▪▪▪	NEW	Unknown	2 (0.43)
33	▪□▪▪▪▪▪▪▪▪▪▪□▪▪▪▪▪▪▪▪▪▪▪▪▪▪▪▪▪▪▪□□□□▪▪▪▪▪▪▪	NEW	Unknown	2 (0.43)
34	▪□▪▪▪▪▪▪▪▪▪▪□▪▪▪▪▪▪▪▪▪▪▪▪▪▪▪▪▪□▪□□□□▪▪▪▪▪▪▪	NEW	Unknown	1 (0.21)
35	▪▪▪▪▪▪▪▪▪▪▪▪▪▪▪▪▪▪▪▪▪▪▪▪▪▪▪▪□□□□□□□□□□□▪▪▪▪	NEW	Unknown	1 (0.21)
36	▪□▪□▪▪▪▪▪▪▪▪▪▪▪▪▪▪▪▪▪▪▪▪▪▪▪▪□□□▪□□□□▪▪▪▪▪▪▪	NEW	Unknown	1 (0.21)
37	▪▪▪▪▪▪▪▪▪▪▪▪▪▪▪▪▪▪▪▪▪▪▪▪▪▪▪▪□▪▪▪□□□□▪▪▪□▪▪▪	NEW	Unknown	1 (0.21)
38	▪▪▪▪▪▪▪▪▪▪▪▪▪▪▪□□▪▪▪▪▪▪▪▪▪▪▪▪▪▪▪□□□□▪▪▪□▪▪▪	NEW	Unknown	1 (0.21)
39	▪▪▪▪▪▪▪▪▪▪▪▪□▪▪▪▪▪▪▪▪▪▪▪▪▪▪▪▪▪□▪□□□□□□□▪▪▪▪	NEW	Unknown	1 (0.21)
40	▪□▪▪▪▪▪▪▪▪□□□□▪▪▪▪▪▪▪▪▪▪▪▪▪▪▪▪▪▪□□□□▪▪▪▪▪▪▪	NEW	Unknown	2 (0.43)
41	▪▪▪▪▪▪□□▪□▪▪▪▪▪▪▪▪▪▪▪□□□□▪▪▪▪▪▪▪□□□□▪▪▪▪▪▪▪	NEW	Unknown	1 (0.21)
42	▪▪□▪▪▪▪▪□▪▪▪▪▪▪□▪▪▪▪▪▪▪▪▪▪▪▪▪▪▪▪□▪□▪▪▪□□□□□	NEW	Unknown	1 (0.21)
43	□□□□□□□□□□□□□□□□□□□□□□□□□□□□□□□□□□▪▪▪▪▪▪▪□□	NEW	Unknown	1 (0.21)
44	□□□□□□□□□□□□□□□□□□□□□□□□□□□□□□□□□□▪▪□□□□□□□	NEW	Unknown	1 (0.21)

a SIT number from the Spoldb4.0 database. SIT, spoligotype international type.

b Spoligotype families as assigned in the Spoldb4.0 database.

c Number of isolates with a common SIT

We used Spoldb4.0 database (an international spoligotype database at the Institute Pasteur de Guadeloupe) to compare the spoligotyping results, and applied the published rules for definition of the Beijing lineage (hybridization to at least three of the spacers 35–43 in the genomic direct-repeat region and absence of hybridization to spacers 1–34) [9]. 445 isolates were assigned to six known spoligotype lineages, whereas 22 isolates could not be matched to any and are thus referred to as “Unknown” (Table 1). The largest spoligotype lineage was the Beijing genotype (87.58%, 409 isolates), most of which (378 isolates) belonged to the classical Beijing genotype with a pattern that depicted the absence of the first 34 spacer oligonucleotides and the presence of spacers 35–43 [9], [11]. The second most common lineage was the T lineage (5.35%, 25 isolates), followed by the H lineage (1.07%, 5 isolates), the MANU family (0.64%, 3 isolates), the U family (0.43%, 2 isolates) and CAS lineage with a single isolate (0.21%).

### 15-locus MIRU-VNTR

By using the 15-locus MIRU-VNTR method previously called VNTR15_China_, the 467 isolates were distributed into 228 genotypes (Table S1). A total of 156 isolates had unique patterns, and the rest belonged to 74 genotypes (2 to 41 isolates per genotype). Using a cut-off value of 70% corresponding to 4 VNTR allelic differences between isolates, and the 467 strains fell into 15 groups. Group XIII corresponded to the CAS lineage, T2 and T3 sublineages belonged to group III and T1 isolates occurred mainly in the group I. The large majority of Beijing family isolates, defined by spoligotyping, were found in group VII-XII, group XV and group VI, The other isolates were scattered, although there remained some aggregation.

**Figure 2:**
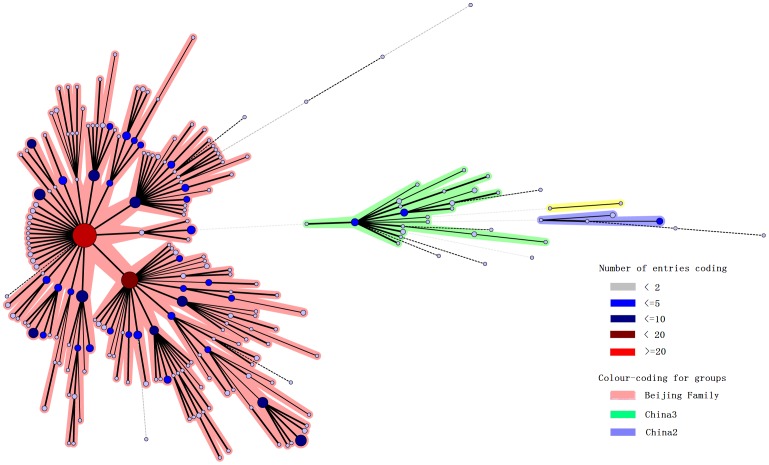
Minimum Spanning tree showing the clustering by VNTR15-_China_ of 467 *M.tuberculosis* isolates from Gansu. Each nodal point represents a cluster with an identical genotypes, the colour and the size of nodal points is relative to the number of strains within that cluster. Different colours are assigned to the different groups, the colour code is indicated on the side.

In Figure 2, the clustering is shown in the form of a minimum spanning tree. The Beijing family isolates form the largest pink group, whereas the other main two groups shown in green and blue (called respectively China2 and China3 in agreement with previous studies [19]) have a spoligotype pattern with absence of S33 to S36. The allelic diversity of each MIRU-VNTR locus for the 467 isolates was estimated by using the Hunter-Gaston discriminatory index (HGDI) (Table 2). The discriminatory power for two loci (Mtub21 and MIRU26) exceeded 0.6, which was considered highly discriminatory [8]. A total of five loci (ETRE, ETRA, MIRU16, MIRU40, and MIRU10) showed moderate discriminatory power (0.3≤h≤0.6). Other loci were less polymorphic, with HGDI within the range of 0–0.3. We also compared the HGDI of these loci with those reported in other areas (Table 3).

**Table 2:** HGDI of the 15 MIRU-VNTR loci for the whole sample and for the Beijing genotype isolates.

	Alias	HGDI score	HGDI score
		All strains	Beijing family
1	Mtub21	0.694	0.653
2	MIRU26	0.627	0.581
3	ETRE	0.511	0.409
4	MIRU16	0.403	0.398
5	ETRA	0.351	0.263
6	MIRU10	0.339	0.230
7	MIRU40	0.335	0.276
8	Mtub30	0.265	0.125
9	Mtub39	0.253	0.199
10	MIRU39	0.242	0.090
11	ETRB	0.170	0.034
12	ETRD	0.116	0.090
13	MIRU27	0.114	0.080
14	MIRU23	0.079	0.057
15	ETRC	0.051	0.039
	Cumulative HGDI	0.986	0.984

HDGI, Hunter-Gaston discriminatory index; MIRU-VNTR, mycobacterial interspersed repetitive units–variable number tandem repeats.

**Table 3:** Allelic diversity of different MIRU-VNTR markers in Beijing family isolates from different locations.

VNTR alias	Gansu, China (this study)	Beijing, China[14]	Heilongjiang,China[22]	Shanghai, China[21]	Tibet, China[16]	Hong Kong, China[23]	St. Petersburg, Russia[24]	Kobe, Japan[20]
ETRA	0.263	0.232	0.238	0.031	0.090	0.201	0.158	0.147
ETRB	0.034	0.014		0	0.029	0	0	0
ETRC	0.039	0.094			0.054	0.165	0.042	0.022
ETRD	0.090	0.120	0.212	0.061	0.066	0.019	0	0.086
ETRE	0.409	0.169	0.395	0.246	0.617	0.200	0.160	0.322
MIRU10	0.230	0.144	0.154	0.195	0.025	0.377	0.082	0.419
MIRU16	0.398	0.068	0.200	0.242	0.158	0.058	0.082	0.310
MIRU23	0.057	0.014		0.061	0.033		0	0.176
MIRU26	0.581	0.353	0.596	0.612	0.429	0.200	0.520	0.383
MIRU27	0.080	0.014		0.031	0.058		0	0.115
MIRU39	0.090	0.119	0.290	0.286	0.147	0.320	0	0.221
MIRU40	0.276	0.194	0.292	0.147	0.221	0.196	0.327	0.122
Mtub21	0.653	0.556	0.396	0.523	0.491		0.330	0.393
Mtub30	0.125	0.068	0.133	0.091	0.033		0.042	0.403
Mtub39	0.199	0.171	0.174	0.061	0.166		0	0.186

### Comparison between Spoligotyping and 15-Loci MIRU-VNTR

As shown in Figure S1, there was some discrepancy between the two methods concerning isolates of the Beijing family. Indeed, inside the largest MIRU-VNTR group, all isolates except 6 showed the characteristic Beijing-family spoligotype. This may be due to the presence of two independent strains in some clinical samples. Combination of spoligotyping and MIRU-VNTR data, 467 strains divided in 251 clusters (with identical genotypes). As displayed in Table 1, 11 spoligotype variants of the Beijing family were detected in the 409 Beijing family isolates, and the HGDI score was 14.43%. Interestingly, as previously shown, the 15-loci MIRU-VNTR analysis defined 193 genotypes, with an HGDI score of 98.38% confirming that VNTR15_China_ method is suitable for typing Beijing strains in China.

### Relationship between the Beijing family and sex, age, and treatment history

We analyzed the relationship between the Beijing genotype and sex, age (>45 and ≤45), and treatment history of patients (new or returning patient), but found no statistically significant associations (*P*>.05 for all) (Table 4).

**Table 4:** Statistical analysis of Beijing genotype and sex, age, and treatment status.

	Beijing genotype	non-Beijing genotype	OR	P	95% CI
All patients	409	58			
Sex					
Male	247	34	1		
Female	162	24	1.153	0.622	0.655–2.028
Age					
0–45	257	35	1		
>45	152	23	1.091	0.765	0.616–1.934
TB treatment history					
New patient	235	34	1		
Repeat treatment	174	24	1.022	0.941	0.581–1.796

CI, confidence interval.

### Relationship between groups and geographic characteristics, sex and age

The 14 regions of the Gansu province were grouped into two different areas, i.e. Northern area and Southern, area according to geographic division (including respectively Jiuquan, Jiayuguan, Zhangye, Jinchang, Wuwei, Baiyin and Lanzhou; and Qingyang, Pingliang, Dingxi, Tianshui, Longnan, Linxia Hui Autonomous Prefecture and Gannan Tibetan Autonomous Prefecture). We then compared the distribution of isolates from the four larger groups (more than five isolates belonging to each groups) in those areas (Table 5). There was no difference in the dendrogram and cluster analysis in different areas, and there were no patient-sex and age differences among the 4 larger groups (*P*>05 for all).

**Table 5:** Comparison of the VNTR15-_China_ groups in different areas in Gansu.

	Group I (mainly T1)	Group III (mainly T2&T3)	Group VI (mainly Beijing family)	Group IX (mainly Beijing family)	P
Sex					
Male	17	6	246	31	
Female	15	3	122	7	0.294
Age					
0–45	18	5	233	23	
>45	14	4	135	15	0.829
Regions					
North area	21	4	246	31	
South area	11	5	1.22	7	0.113

## Discussion

To the best of our knowledge, this is the first detailed study on the genetic diversity of *M. tuberculosis* in Gansu Province using two genotyping approaches. The results indicate that the population structure of *M. tuberculosis* isolates in Gansu appears to be very homogeneous, as six spoligotype lineages were obtained from the 467 isolates, with 87.58% of the isolates belonging to the Beijing genotype. The predominance of the Beijing genotype in China has been well described. It is prevalent in Beijing (80–92.6%) [11], [14], [15], Tianjin (91.7%) [12], Jilin (89.9%) [15], Tibet (96.3%) [15], [16], and Inner Mongolia (93.26%) [10], but less prevalent in areas of southern China, including Guangdong (25%) [13], Guangxi (55.3%), and Fujian (54.5%) [15]. Hence, Gansu is one of the regions in which the proportion of the Beijing genotype is high. At the same time, due to the unique geography of Gansu Province, a wide range of genotypes might be linked with a huge number of floating populations.

In this study, there was no association between the prevalence of the Beijing genotype and gender and treatment status. An epidemiological investigation in Gansu Province showed that the patient age of 45 years was a special point of active TB, as the prevalence of smear- and culture-positive TB in patients >45 years old was higher than that in patients <45 years old [32]. Therefore, we tried to figure out whether there is a difference in prevalence of the Beijing genotype between the two age groups. However, in our study, the Beijing genotype was the main genotype in both age groups. This finding indicated that there might be other factors that affected the spread of Beijing genotype strains. Some researchers think that, based on co-evolution between the host and the pathogen, demographic factors may be responsible for the dominance of Beijing genotype strains [17], [18]. This estimation for the correlation between the Beijing genotype and sex, treatment status and age were, perhaps, biased by a smaller sample size of non-Beijing genotype strains compared to Beijing genotype strains.

Due to the low discriminatory power of the spoligotyping method for Beijing genotype strains, these strains were further subjected to the VNTR15_China_ method, and we found that the allelic diversity of the VNTR loci varied significantly at each locus, as previously observed [19]. Among the 15 loci, Mtub21 and MIRU26 were highly discriminative (h≥0.6), ETRE, ETRA, MIRU16, MIRU40, and MIRU10 were moderately discriminative, while other loci were less polymorphic. When comparing the allelic diversity among all strains, and among Beijing genotype strains, all 15 MIRU-VNTR loci and cumulative HGDI score showed lower allelic diversity among Beijing genotype strains and were consistent with the close genetic relationships of those strains, as was also showed in previous studies [10], [14], [16]. By comparison with results obtained in other geographical areas, we found that the HGDI of ETR-B was 0.034 in Gansu, and 0 in Hongkong, St. Petersburg and Japan. Similarly, MIRU23, MIRU27 and MIRU39 also displayed no allelic diversity in St. Petersburg, although they showed some polymorphism in Gansu. Four loci in VNTR15_China_ do not belong to the standard 15-loci VNTR subset [34] and present a rather low level of allelic diversity. We retained them in our scheme because they are useful to anchor the different lineages [35]. In addition, these 4 loci appeared to be suitable for typing the China strains, compared to the results described in previous reports [20], [24]. MIRU26, a highly discriminative locus in Gansu and Shanghai, showed a lower discriminative power in Hongkong, whereas Mtub21 was polymorphic in Gansu isolates, but was moderately discriminative in isolates from other areas [14], [16], [20], [21], [22], [23], [24]. Although we found that MIRU-VNTR loci showed variable abilities to differentiate Beijing genotype strains from different areas, this may be attributed to the dissimilarities in the population structures of the circulating *M. tuberculosis* strains in distinct areas [35]. We have compared the present data from Gansu to those from other provinces, previously typed with the same MIRU-VNTR scheme [19] and did not find a particular distribution. This suggests that the diversity in Gansu is related to movements of population rather than to a local evolution.

The results obtained on the basis of VNTR15_China_ typing were slightly different with those of spoligotyping, but this may be due to the presence of two different isolates in some samples. The Beijing genotype strains aggregated into several MIRU-VNTR groups, the largest being group VI. We analysed the relationship between the 4 larger groups and strains' distribution, sex or age, but we found no correlation between them. The smaller groups were not considered in this analysis because it would not be statistically relevant. Although spoligotyping yielded the most unambiguous identification of Beijing genotype strains in a fastest and easiest way, MIRU-VNTR typing had a better discriminatory power for these strains.

In conclusion, the Beijing family is the predominant *M. tuberculosis* lineage in Gansu Province. The existence of a wide range of genotypes inside this lineage might be the consequence of frequent movements of the population. Among the non-Beijing isolates, 18 samples showed a previously unknown spoligotype and some are grouped by MIRU-VNTR analysis. They may represent a characteristic lineage of Gansu. The analysis of MIRU-VNTR data is useful for selecting the appropriate VNTR loci for the genotyping of *M. tuberculosis* in specific geographical areas. Combined with spoligotyping, it represents the most suitable approach for genotyping analysis of *M. tuberculosis* in China.

## Materials and Methods

### Ethics statement

This research was approved by the Ethics Committee of the National Institute for Communicable Disease Control and Prevention, Chinese Center for Disease Control and Prevention. The patients included in this study provided written informed consent before participating.

### Study population and bacterial strains

The study included 467 *M. tuberculosis* isolates, randomly collected from sputum samples of 467 pulmonary TB confirmed patients in TB hospitals, as well as institutes for TB control. They were recovered between 2005 and 2011 in Gansu Province, China, predominantly from the province's TB hospital (Lanzhou Chest Hospital). This hospital cares mainly for TB patients who are coming from all over the province. All isolates were stored at -70°C, and recovered on Lowenstein-Jensen (L-J) medium for 28 days at 37°C when needed. Chromosomal DNA was prepared by boiling a loopful of colonies from L-J slants in 400 µL of 10 mM Tris-HCL and 1 mM EDTA (pH 8.0) buffer for 10 minutes. The suspension was centrifuged at 12,000 rpm for 10 minutes, the cell debris was removed, and the lysates were stored at −20°C until use [25].

### PCR amplification

5 µL of lysates obtained from the cultured *M. tuberculosis* strain was added to 35 µL of the PCR mixture. The PCR amplification cycles consisted of the following: 5 minutes at 94°C for DNA denaturation; 35 cycles of 45 seconds at 94°C for DNA denaturation, 45 seconds at 62°C for primer annealing, and 1 minute at 72°C for primer extension; and a last cycle of 10 minutes at 72°C for primer extension.

### MIRU-VNTR genotyping

We used the VNTR15-_China_ Scheme [19] comprising the following markers: ETR-A, ETR-B, ETR-C, ETR-D (MIRU04), ETR-E (MIRU31), MIRU10, MIRU16, MIRU23, MIRU26, MIRU27, MIRU39, MIRU40, Mtub21, Mtub30, and Mtub39. Each MIRU-VNTR locus was amplified individually in reaction volume, and electrophoresis of products on agarose gels was carried out as described in a previous report [26].

### Spoligotyping

Spoligotyping of the isolates was performed as described by Kamerbeek et al [27]. The direct repeat (DR) region was amplified with the primer pair in reaction volume, and the PCR products were hybridized to a set of 43 oligonucleotide probes corresponding to each spacer that were covalently bound to a membrane. Spoligotypes in binary formats were compared with the Spoldb4.0 database, an updated version of the published fourth international spoligotyping database [28].

### Data management and analysis

The gel images were analyzed using the BioNumerics software package (version 6.5; Applied-Maths, Sint-Martens-Latem, Belgium) as previously described [26], [29]. Clustering analysis was done using the unweighted pair group method with arithmetic averages. The dice and categorical coefficients were used in spoligotyping and MIRU-VNTR, respectively. Discrimination of the locus combination was calculated using the HDGI [30]:
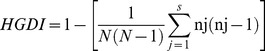
where *N* is the total number of isolates in the typing method, *s* is the number of distinct patterns discriminated by MIRU-VNTR, and *n_j_* is the number of isolates belonging to the *j*th pattern. The clustering rate was defined as *(n_c_-c)/n*, whereby *n_c_* is the total number of clustered cases, *c* is the number of clusters, and *n* is the total number of isolates [31].

Heterogeneity of sex, age, and treatment status in the Beijing and non-Beijing genotypes was assessed using the *chi-square* test using SPSS 19.0 (SPSS Inc., Chicago, IL, USA). Values of *p*<0.05 were considered statistically significant.

## Supporting Information

Figure S1 **Genotyping of 467** ***M. tuberculosis*** **isolates with VNTR15-_China_ and Spoligotyping.** The clustering was based on the analysis performed using BioNumerics 6.5 to compare these two genotyping methods. From left to right: 1) UPGMA dendrogram generated by VNTR15-_China_ 2) the repeat number in each VNTR-locus 3) spoligotyping patterns 4) strain No.
(PDF)

Table S1 **The 15-locus MIRU-VNTR repeats profiles, and spoligotyping profiles of the 467 isolates.**
(XLS)
