# Supplementary material for: First Insight into the Genotypic Diversity of Clinical Mycobacterium tuberculosis Isolates from Gansu Province, China
Source: PLoS One. 2014 Jun 9;9(6):e99357. doi: 10.1371/journal.pone.0099357 (PMC4049826; doi:10.1371/journal.pone.0099357)

Categorical
vntr

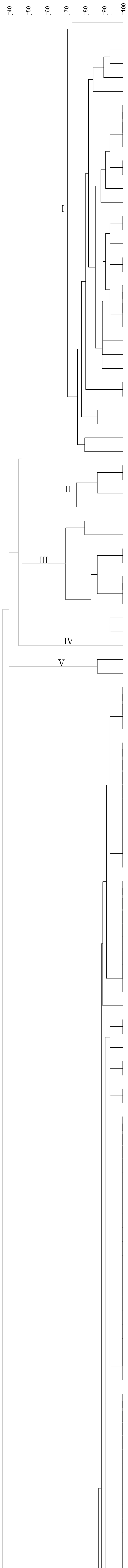

vntr

ETRA	ETRB	ETRC	ETRD	ETRE	MIRU10	MIRU16	MIRU23	MIRU26	MIRU27	MIRU39	MIRU40	Mtub21	Mtub30	Mtub39	Strain
2	1	4	3	3	2	2	5	5	3	2	1	3	2	6	GS05040
3	1	4	3	3	2	3	5	4	4	2	1	3	2	6	GS12497
3	1	4	3	3	2	2	5	5	3	2	3	3	2	4	GS06036
3	1	4	3	2	2	2	5	5	3	2	3	3	2	4	GS07009
3	1	4	3	2	2	1	5	5	3	2	3	3	2	4	GS06088
3	1	4	3	3	2	1	5	5	3	2	1	3	2	4	GS12132
3	1	4	3	3	2	3	5	5	3	2	4	3	2	4	GS12350
3	1	4	3	3	2	3	5	5	3	2	4	3	2	4	GS12352
3	1	4	3	3	2	3	5	5	3	2	4	3	2	4	GS12429
3	1	4	3	3	2	3	5	5	3	2	4	3	2	4	GS12439
2	1	4	3	3	2	3	5	5	3	2	4	3	2	4	GS12307
2	1	4	3	3	2	3	5	5	3	2	4	3	2	4	GS12415
3	1	4	3	3	2	3	5	3	3	2	4	3	2	4	GS05115
2	1	4	3	3	2	3	5	4	3	2	4	3	2	4	GS12506
3	1	4	3	3	2	3	5	6	3	2	3	3	2	4	GS06096
3	1	4	3	3	2	3	5	6	3	2	3	3	2	4	GS07013
3	1	4	3	3	2	3	5	7	3	2	3	3	2	4	GS12392
3	1	4	3	3	2	3	5	5	3	2	3	3	2	3	GS12372
3	1	4	3	3	2	3	5	5	3	2	3	3	2	3	GS12393
3	1	4	3	3	2	3	5	5	3	2	3	3	2	4	GS07057
3	1	4	3	3	2	3	5	5	3	2	3	3	2	4	GS12035
3	1	4	3	3	2	3	5	5	3	2	3	3	2	4	GS12233
3	1	4	3	3	2	3	5	5	3	2	3	3	2	4	GS12237
3	1	4	2	3	2	3	5	5	3	2	3	3	2	4	GS07018
3	1	4	3	3	2	3	5	5	4	2	3	3	2	4	GS07029
2	1	4	3	3	2	3	5	5	3	2	3	3	2	4	GS12499
3	1	4	3	3	2	2	5	6	3	2	4	3	2	4	GS06006
3	1	4	3	3	2	2	5	6	3	2	4	3	2	4	GS06007
3	1	4	3	3	2	3	5	5	2	2	3	2	2	4	GS12383
3	1	5	3	3	2	3	5	5	2	2	3	3	2	4	GS12453
3	1	4	4	3	2	3	5	6	3	2	5	3	2	4	GS12225
3	1	4	3	3	2	3	5	4	3	2	5	3	2	5	GS12440
2	1	4	3	3	2	3	4	5	3	2	3	3	2	5	GS05132
2	1	4	3	3	2	3	4	5	3	2	3	3	2	5	GS05154
2	1	4	3	3	3	3	4	5	2	2	3	3	2	5	GS05163
2	1	4	3	2	2	3	6	5	3	2	3	3	2	3	GS12468
4	2	3	5	3	2	2	6	5	2	2	2	1	2	2	GS05023
4	2	3	5	3	3	3	6	5	3	2	2	1	2	2	GS05160
4	2	4	8	3	2	3	5	5	2	2	2	1	2	2	GS12017
4	2	4	8	3	2	3	5	5	2	2	2	1	2	2	GS12023
4	2	4	3	3	2	2	5	5	2	2	2	1	2	2	GS06049
4	2	4	3	3	2	2	5	5	2	2	2	1	2	2	GS06051
4	2	4	3	3	2	2	5	5	2	2	2	1	2	2	GS06053
3	2	4	5	3	2	2	5	5	2	2	2	1	2	2	GS05056
3	2	4	5	3	2	3	5	5	2	2	2	1	2	2	GS07043
5	4	5	3	3	2	3	4	4	3	2	2	1	2	3	GS12058
3	3	4	3	3	3	1	6	3	3	2	1	2	2	6	GS05071
3	3	4	4	3	3	2	6	3	3	2	1	2	2	6	GS12076
4	2	4	3	4	3	3	5	6	3	3	3	5	4	4	GS07055
4	2	4	3	4	3	3	5	6	3	3	3	5	4	4	GS12316
4	2	4	3	4	3	3	5	6	3	3	3	5	4	4	GS12426
4	2	4	3	4	3	3	5	6	3	3	3	5	4	4	GS12560
4	2	4	3	5	3	3	5	6	3	3	3	5	4	4	GS05140
4	2	4	3	5	3	3	5	6	3	3	3	5	4	4	GS12095
4	2	4	3	5	3	3	5	6	3	3	3	5	4	4	GS12183
4	2	4	3	5	3	3	5	6	3	3	3	5	4	4	GS12229
4	2	4	3	5	3	3	5	6	3	3	3	5	4	4	GS12460
4	2	4	3	5	3	3	5	6	3	3	3	5	4	4	GS12568
4	2	4	3	5	3	3	5	6	3	3	3	5	4	4	GS12573
4	2	4	3	5	3	3	5	6	3	3	3	5	4	4	GS12586
4	2	4	3	5	3	3	5	6	3	3	3	5	4	4	GS12587
4	2	4	3	5	3	3	5	6	3	3	3	5	4	4	GS12592
4	2	4	3	5	3	3	5	5	3	3	3	5	4	4	GS05172
4	2	4	3	5	3	3	5	5	3	3	3	5	4	4	GS07052
4	2	4	3	5	3	3	5	5	3	3	3	5	4	4	GS12062
4	2	4	3	5	3	3	5	5	3	3	3	5	4	4	GS12191
4	2	4	3	5	3	3	5	5	3	3	3	5	4	4	GS12390
4	2	4	3	5	3	3	5	5	3	3	3	5	4	4	GS12432
4	2	4	3	5	3	3	5	5	3	3	3	5	4	4	GS12582
4	2	4	3	5	3	3	5	5	3	3	3	5	4	4	GS12589
4	2	4	3	5	3	3	5	5	3	3	3	5	4	4	GS12594
4	2	4	3	5	3	3	5	6	3	3	3	5	2	4	GS12146
4	3	4	3	5	3	3	5	7	3	3	3	5	4	4	GS07039
4	3	4	3	5	3	3	5	7	3	3	3	5	4	4	GS07045
4	1	4	3	5	3	3	5	7	3	3	3	5	4	4	GS12027
4	2	4	3	5	3	3	5	7	3	3	3	3	4	4	GS12482
4	2	4	3	5	3	3	5	7	3	3	3	3	4	4	GS12579
4	2	4	3	5	3	3	5	7	3	3	3	8	4	4	GS12134
4	2	4	3	5	3	3	5	7	3	3	3	8	4	4	GS12219
4	2	4	3	5	3	3	5	7	3	3	3	4	4	4	GS05092
4	2	4	3	5	3	3	5	7	3	3	3	4	4	4	GS05106
4	2	4	3	5	3	3	5	7	3	3	3	4	4	4	GS05112
4	2	4	3	5	3	3	5	7	3	3	3	4	4	4	GS05156
4	2	4	3	5	3	3	5	7	3	3	3	4	4	4	GS05188
4	2	4	3	5	3	3	5	7	3	3	3	4	4	4	GS05197
4	2	4	3	5	3	3	5	7	3	3	3	4	4	4	GS07008
4	2	4	3	5	3	3	5	7	3	3	3	4	4	4	GS07030
4	2	4	3	5	3	3	5	7	3	3	3	4	4	4	GS07035
4	2	4	3	5	3	3	5	7	3	3	3	4	4	4	GS12041
4	2	4	3	5	3	3	5	7	3	3	3	4	4	4	GS12055
4	2	4	3	5	3	3	5	7	3	3	3	4	4	4	GS12118
4	2	4	3	5	3	3	5	7	3	3	3	4	4	4	GS12317
4	2	4	3	5	3	3	5	7	3	3	3	4	4	4	GS12339
4	2	4	3	5	3	3	5	7	3	3	3	4	4	4	GS12427
4	2	4	3	5	3	3	5	7	3	3	3	4	4	4	GS12500
4	2	4	3	5	3	3	5	7	3	3	3	4	4	4	GS12504
4	2	4	3	5	3	3	5	7	3	3	3	4	4	4	GS12514
4	2	4	3	5	3	3	5	7	3	3	3	4	4	4	GS12537
4	2	4	3	5	3	3	5	7	3	3	3	4	4	4	GS12598
4	2	4	3	5	3	3	5	7	3	3	3	5	4	4	GS05081
4	2	4	3	5	3	3	5	7	3	3	3	5	4	4	GS05179
4	2	4	3	5	3	3	5	7	3	3	3	5	4	4	GS07007
4	2	4	3	5	3	3	5	7	3	3	3	5	4	4	GS07032
4	2	4	3	5	3	3	5	7	3	3	3	5	4	4	GS07050
4	2	4	3	5	3	3	5	7	3	3	3	5	4	4	GS07056
4	2	4	3	5	3	3	5	7	3	3	3	5	4	4	GS07059
4	2	4	3	5	3	3	5	7	3	3	3	5	4	4	GS12002
4	2	4	3	5	3	3	5	7	3	3	3	5	4	4	GS12005
4	2	4	3	5	3	3	5	7	3	3	3	5	4	4	GS12036
4	2	4	3	5	3	3	5	7	3	3	3	5	4	4	GS12037
4	2	4	3	5	3	3	5	7	3	3	3	5	4	4	GS12045
4	2	4	3	5	3	3	5	7	3	3	3	5	4	4	GS12047

sp

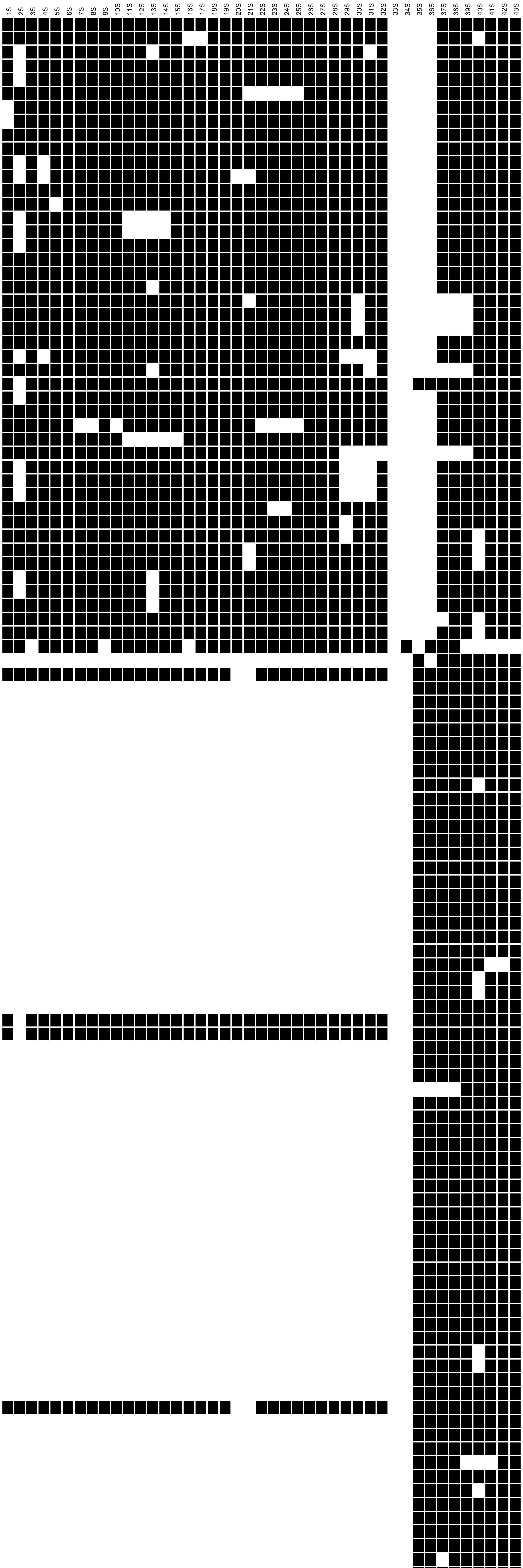

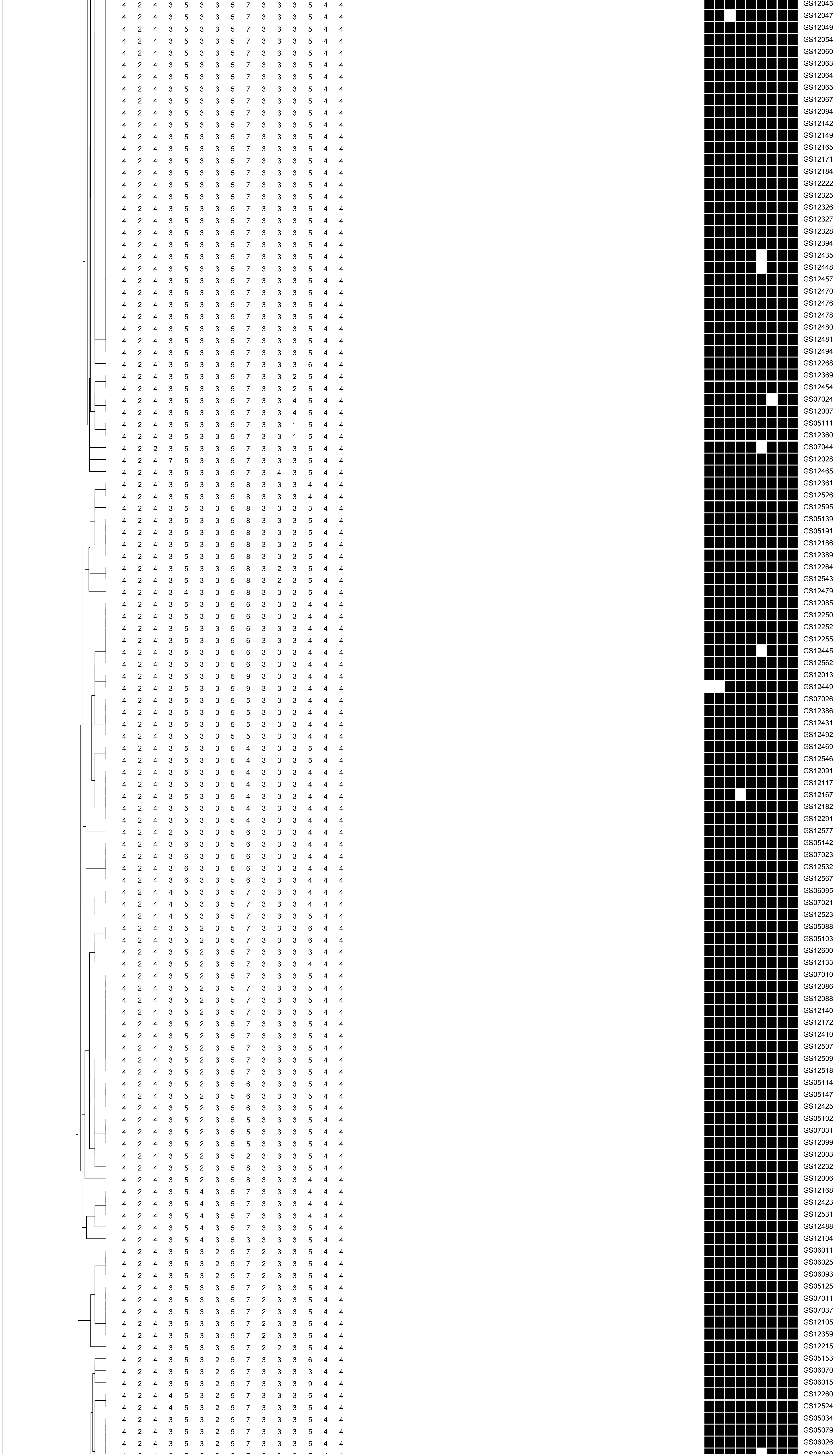
4 2 4 3 5 3 3 5 7 3 3 3 5 4 4 GS12045
4 2 4 3 5 3 3 5 7 3 3 3 5 4 4 GS12047
4 2 4 3 5 3 3 5 7 3 3 3 5 4 4 GS12049
4 2 4 3 5 3 3 5 7 3 3 3 5 4 4 GS12054
4 2 4 3 5 3 3 5 7 3 3 3 5 4 4 GS12060
4 2 4 3 5 3 3 5 7 3 3 3 5 4 4 GS12063
4 2 4 3 5 3 3 5 7 3 3 3 5 4 4 GS12064
4 2 4 3 5 3 3 5 7 3 3 3 5 4 4 GS12065
4 2 4 3 5 3 3 5 7 3 3 3 5 4 4 GS12067
4 2 4 3 5 3 3 5 7 3 3 3 5 4 4 GS12094
4 2 4 3 5 3 3 5 7 3 3 3 5 4 4 GS12142
4 2 4 3 5 3 3 5 7 3 3 3 5 4 4 GS12149
4 2 4 3 5 3 3 5 7 3 3 3 5 4 4 GS12165
4 2 4 3 5 3 3 5 7 3 3 3 5 4 4 GS12171
4 2 4 3 5 3 3 5 7 3 3 3 5 4 4 GS12184
4 2 4 3 5 3 3 5 7 3 3 3 5 4 4 GS12222
4 2 4 3 5 3 3 5 7 3 3 3 5 4 4 GS12325
4 2 4 3 5 3 3 5 7 3 3 3 5 4 4 GS12326
4 2 4 3 5 3 3 5 7 3 3 3 5 4 4 GS12327
4 2 4 3 5 3 3 5 7 3 3 3 5 4 4 GS12328
4 2 4 3 5 3 3 5 7 3 3 3 5 4 4 GS12394
4 2 4 3 5 3 3 5 7 3 3 3 5 4 4 GS12435
4 2 4 3 5 3 3 5 7 3 3 3 5 4 4 GS12448
4 2 4 3 5 3 3 5 7 3 3 3 5 4 4 GS12457
4 2 4 3 5 3 3 5 7 3 3 3 5 4 4 GS12470
4 2 4 3 5 3 3 5 7 3 3 3 5 4 4 GS12476
4 2 4 3 5 3 3 5 7 3 3 3 5 4 4 GS12478
4 2 4 3 5 3 3 5 7 3 3 3 5 4 4 GS12480
4 2 4 3 5 3 3 5 7 3 3 3 5 4 4 GS12481
4 2 4 3 5 3 3 5 7 3 3 3 5 4 4 GS12494
4 2 4 3 5 3 3 5 7 3 3 3 6 4 4 GS12268
4 2 4 3 5 3 3 5 7 3 3 2 5 4 4 GS12369
4 2 4 3 5 3 3 5 7 3 3 2 5 4 4 GS12454
4 2 4 3 5 3 3 5 7 3 3 4 5 4 4 GS07024
4 2 4 3 5 3 3 5 7 3 3 4 5 4 4 GS12007
4 2 4 3 5 3 3 5 7 3 3 1 5 4 4 GS05111
4 2 4 3 5 3 3 5 7 3 3 1 5 4 4 GS12360
4 2 2 3 5 3 3 5 7 3 3 3 5 4 4 GS07044
4 2 4 7 5 3 3 5 7 3 3 3 5 4 4 GS12028
4 2 4 3 5 3 3 5 7 3 4 3 5 4 4 GS12465
4 2 4 3 5 3 3 5 8 3 3 3 4 4 4 GS12361
4 2 4 3 5 3 3 5 8 3 3 3 4 4 4 GS12526
4 2 4 3 5 3 3 5 8 3 3 3 3 4 4 GS12595
4 2 4 3 5 3 3 5 8 3 3 3 5 4 4 GS05139
4 2 4 3 5 3 3 5 8 3 3 3 5 4 4 GS05191
4 2 4 3 5 3 3 5 8 3 3 3 5 4 4 GS12186
4 2 4 3 5 3 3 5 8 3 3 3 5 4 4 GS12389
4 2 4 3 5 3 3 5 8 3 2 3 5 4 4 GS12264
4 2 4 3 5 3 3 5 8 3 2 3 5 4 4 GS12543
4 2 4 3 4 3 3 5 8 3 3 3 5 4 4 GS12479
4 2 4 3 5 3 3 5 6 3 3 3 4 4 4 GS12085
4 2 4 3 5 3 3 5 6 3 3 3 4 4 4 GS12250
4 2 4 3 5 3 3 5 6 3 3 3 4 4 4 GS12252
4 2 4 3 5 3 3 5 6 3 3 3 4 4 4 GS12255
4 2 4 3 5 3 3 5 6 3 3 3 4 4 4 GS12445
4 2 4 3 5 3 3 5 6 3 3 3 4 4 4 GS12562
4 2 4 3 5 3 3 5 9 3 3 3 4 4 4 GS12013
4 2 4 3 5 3 3 5 9 3 3 3 4 4 4 GS12449
4 2 4 3 5 3 3 5 5 3 3 3 4 4 4 GS07026
4 2 4 3 5 3 3 5 5 3 3 3 4 4 4 GS12386
4 2 4 3 5 3 3 5 5 3 3 3 4 4 4 GS12431
4 2 4 3 5 3 3 5 5 3 3 3 4 4 4 GS12492
4 2 4 3 5 3 3 5 4 3 3 3 5 4 4 GS12469
4 2 4 3 5 3 3 5 4 3 3 3 5 4 4 GS12546
4 2 4 3 5 3 3 5 4 3 3 3 4 4 4 GS12091
4 2 4 3 5 3 3 5 4 3 3 3 4 4 4 GS12117
4 2 4 3 5 3 3 5 4 3 3 3 4 4 4 GS12167
4 2 4 3 5 3 3 5 4 3 3 3 4 4 4 GS12182
4 2 4 3 5 3 3 5 4 3 3 3 4 4 4 GS12291
4 2 4 2 5 3 3 5 6 3 3 3 4 4 4 GS12577
4 2 4 3 6 3 3 5 6 3 3 3 4 4 4 GS05142
4 2 4 3 6 3 3 5 6 3 3 3 4 4 4 GS07023
4 2 4 3 6 3 3 5 6 3 3 3 4 4 4 GS12532
4 2 4 3 6 3 3 5 6 3 3 3 4 4 4 GS12567
4 2 4 4 5 3 3 5 7 3 3 3 4 4 4 GS06095
4 2 4 4 5 3 3 5 7 3 3 3 4 4 4 GS07021
4 2 4 4 5 3 3 5 7 3 3 3 5 4 4 GS12523
4 2 4 3 5 2 3 5 7 3 3 3 6 4 4 GS05088
4 2 4 3 5 2 3 5 7 3 3 3 6 4 4 GS05103
4 2 4 3 5 2 3 5 7 3 3 3 3 4 4 GS12600
4 2 4 3 5 2 3 5 7 3 3 3 4 4 4 GS12133
4 2 4 3 5 2 3 5 7 3 3 3 5 4 4 GS07010
4 2 4 3 5 2 3 5 7 3 3 3 5 4 4 GS12086
4 2 4 3 5 2 3 5 7 3 3 3 5 4 4 GS12088
4 2 4 3 5 2 3 5 7 3 3 3 5 4 4 GS12140
4 2 4 3 5 2 3 5 7 3 3 3 5 4 4 GS12172
4 2 4 3 5 2 3 5 7 3 3 3 5 4 4 GS12410
4 2 4 3 5 2 3 5 7 3 3 3 5 4 4 GS12507
4 2 4 3 5 2 3 5 7 3 3 3 5 4 4 GS12509
4 2 4 3 5 2 3 5 7 3 3 3 5 4 4 GS12518
4 2 4 3 5 2 3 5 6 3 3 3 5 4 4 GS05114
4 2 4 3 5 2 3 5 6 3 3 3 5 4 4 GS05147
4 2 4 3 5 2 3 5 6 3 3 3 5 4 4 GS12425
4 2 4 3 5 2 3 5 5 3 3 3 5 4 4 GS05102
4 2 4 3 5 2 3 5 5 3 3 3 5 4 4 GS07031
4 2 4 3 5 2 3 5 5 3 3 3 5 4 4 GS12099
4 2 4 3 5 2 3 5 2 3 3 3 5 4 4 GS12003
4 2 4 3 5 2 3 5 8 3 3 3 5 4 4 GS12232
4 2 4 3 5 2 3 5 8 3 3 3 4 4 4 GS12006
4 2 4 3 5 4 3 5 7 3 3 3 4 4 4 GS12168
4 2 4 3 5 4 3 5 7 3 3 3 4 4 4 GS12423
4 2 4 3 5 4 3 5 7 3 3 3 4 4 4 GS12531
4 2 4 3 5 4 3 5 7 3 3 3 5 4 4 GS12488
4 2 4 3 5 4 3 5 3 3 3 3 5 4 4 GS12104
4 2 4 3 5 3 2 5 7 2 3 3 5 4 4 GS06011
4 2 4 3 5 3 2 5 7 2 3 3 5 4 4 GS06025
4 2 4 3 5 3 2 5 7 2 3 3 5 4 4 GS06093
4 2 4 3 5 3 3 5 7 2 3 3 5 4 4 GS05125
4 2 4 3 5 3 3 5 7 2 3 3 5 4 4 GS07011
4 2 4 3 5 3 3 5 7 2 3 3 5 4 4 GS07037
4 2 4 3 5 3 3 5 7 2 3 3 5 4 4 GS12105
4 2 4 3 5 3 3 5 7 2 3 3 5 4 4 GS12359
4 2 4 3 5 3 3 5 7 2 2 3 5 4 4 GS12215
4 2 4 3 5 3 2 5 7 3 3 3 6 4 4 GS05153
4 2 4 3 5 3 2 5 7 3 3 3 3 4 4 GS06070
4 2 4 3 5 3 2 5 7 3 3 3 9 4 4 GS06015
4 2 4 4 5 3 2 5 7 3 3 3 5 4 4 GS12260
4 2 4 4 5 3 2 5 7 3 3 3 5 4 4 GS12524
4 2 4 3 5 3 2 5 7 3 3 3 5 4 4 GS05034
4 2 4 3 5 3 2 5 7 3 3 3 5 4 4 GS05079
4 2 4 3 5 3 2 5 7 3 3 3 5 4 4 GS06026

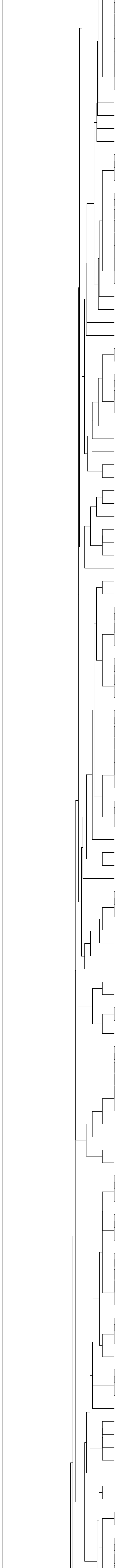

4	2	4	3	5	3	2	5	7	3	3	3	5	4	4	[illegible]
4	2	4	3	5	3	2	5	7	3	3	3	5	4	4	GS06026
4	2	4	3	5	3	2	5	7	3	3	3	5	4	4	GS06060
4	2	4	3	5	3	2	5	7	3	3	3	5	4	4	GS06063
4	2	4	3	5	3	2	5	7	3	3	3	5	4	4	GS06071
4	2	4	3	5	3	2	5	7	3	3	3	5	4	4	GS06086
4	2	4	3	5	3	2	5	7	3	3	3	5	4	4	GS06104
4	2	4	3	5	3	2	5	7	3	3	3	5	4	4	GS06106
4	2	4	3	5	3	2	5	7	3	3	2	5	4	4	GS05054
4	2	4	3	5	3	2	5	7	3	3	3	5	4	2	GS06024
4	2	4	3	8	3	2	5	7	3	3	3	5	4	4	GS06065
4	2	4	3	5	2	2	5	7	3	3	3	5	4	4	GS06089
4	2	4	3	5	3	4	5	7	3	3	3	4	4	4	GS05121
4	2	4	3	5	3	4	5	7	3	3	3	4	4	4	GS05158
4	2	4	3	5	3	4	5	7	3	3	3	4	4	4	GS05159
4	2	4	3	5	3	2	5	7	3	3	3	4	4	4	GS05036
4	2	4	3	5	3	2	5	7	3	3	3	4	4	4	GS06016
4	2	4	3	5	3	2	5	7	3	3	3	4	4	4	GS06019
4	2	4	3	5	3	2	5	7	3	3	3	4	4	4	GS06023
4	2	4	3	5	3	2	5	7	3	3	3	4	4	4	GS06038
4	2	4	3	5	3	2	5	7	3	3	3	4	4	4	GS06054
4	2	4	3	5	3	2	5	7	3	3	3	4	4	4	GS06074
4	2	4	3	5	3	2	5	7	3	3	3	4	4	4	GS06087
4	2	4	3	5	3	2	5	6	3	3	3	4	4	4	GS06105
4	2	4	3	5	1	2	5	7	3	3	3	4	4	4	GS06018
4	2	5	3	5	3	2	5	7	3	3	3	3	4	4	GS06037
4	2	4	3	5	3	2	5	7	1	3	3	8	4	4	GS06075
4	2	4	3	5	2	2	5	6	3	3	3	5	4	4	GS05029
4	2	4	3	5	2	2	5	6	3	3	3	5	4	4	GS06083
4	2	4	3	5	3	2	5	6	3	3	3	5	4	4	GS05018
4	2	4	3	5	3	2	5	6	3	3	3	5	4	4	GS05022
4	2	4	3	5	3	2	5	6	3	3	3	5	4	4	GS05031
4	2	4	3	5	3	2	5	6	3	3	3	5	4	4	GS06050
4	2	4	3	5	3	2	5	6	3	3	3	5	4	6	GS06094
4	2	4	3	5	2	2	5	6	3	3	3	3	4	4	GS06085
4	2	4	3	5	2	2	4	6	3	3	3	5	4	4	GS05058
4	2	4	3	6	3	2	5	8	3	3	3	5	4	4	GS05030
4	2	4	3	5	3	2	5	8	3	3	3	5	4	4	GS06021
4	2	4	3	5	3	3	5	6	3	3	3	6	4	6	GS12024
4	2	4	3	5	3	3	5	6	3	3	3	6	4	4	GS12575
4	2	4	3	5	3	3	5	6	3	3	3	1	4	4	GS12249
4	2	4	3	7	3	3	5	6	3	3	3	3	4	4	GS05116
4	2	4	3	4	3	3	5	6	3	3	3	3	4	4	GS12442
4	2	4	3	5	3	3	5	6	3	3	3	3	4	4	GS12578
4	2	5	3	5	3	3	5	6	3	3	3	11	4	4	GS12559
4	2	4	3	4	3	2	5	7	3	3	3	8	4	4	GS05069
4	2	4	3	4	3	3	5	7	3	3	3	8	4	4	GS05155
4	2	4	3	4	3	2	5	7	3	3	3	5	4	4	GS12555
4	2	4	3	4	3	2	5	7	3	3	3	5	4	4	GS12556
4	2	4	3	4	3	2	5	7	3	3	3	5	4	4	GS12557
4	2	4	3	4	3	2	5	7	3	3	3	5	4	4	GS12558
4	2	4	3	4	3	3	5	7	3	3	3	5	4	4	GS12097
4	2	4	3	4	3	3	5	7	3	3	3	5	4	4	GS12290
4	2	4	3	4	3	3	5	7	3	3	3	5	4	4	GS12422
4	2	4	3	4	3	3	5	7	3	3	3	5	4	4	GS12574
4	2	4	3	4	3	3	5	7	3	3	3	5	4	6	GS12015
4	2	4	3	4	3	3	5	7	3	3	3	5	4	6	GS12181
4	2	4	3	4	3	3	5	7	3	3	3	5	4	6	GS12197
4	2	4	3	4	3	3	5	7	3	3	3	5	4	6	GS12201
4	2	4	3	4	3	3	5	7	3	3	3	5	4	6	GS12203
4	2	4	3	4	3	3	5	7	3	3	3	5	4	6	GS12401
4	2	4	3	4	3	3	5	7	3	3	3	5	4	6	GS12599
4	2	4	3	4	3	3	5	7	3	3	3	5	4	5	GS05100
4	2	4	3	4	3	3	5	7	3	3	3	5	4	5	GS05101
4	2	4	3	4	3	3	5	7	3	3	3	5	4	5	GS12162
4	2	4	3	4	3	3	5	7	3	3	5	5	4	4	GS12367
5	2	4	3	5	3	3	5	7	3	3	3	5	4	4	GS05135
5	2	4	3	4	3	3	5	7	3	3	3	5	4	4	GS05183
4	2	4	3	4	3	2	5	7	3	3	3	6	4	6	GS12145
4	2	4	3	6	3	3	5	7	3	3	3	5	4	4	GS12046
4	2	4	3	6	3	3	5	7	3	3	3	5	4	4	GS12051
4	2	4	3	6	3	3	5	7	3	3	3	5	4	4	GS12414
4	2	4	3	6	3	3	5	7	3	3	3	6	4	4	GS12437
4	2	4	3	6	3	3	5	7	3	2	3	5	4	4	GS07040
4	2	4	3	6	3	3	6	7	3	3	3	6	4	4	GS12544
4	2	5	3	6	3	3	5	7	3	1	3	5	4	4	GS12355
4	2	4	3	5	3	3	5	7	3	3	3	4	4	6	GS12032
3	2	4	3	5	3	3	5	7	3	3	3	4	4	6	GS12570
2	2	4	3	5	3	3	5	7	3	3	3	5	4	6	GS12540
2	2	4	3	5	3	3	5	7	3	3	3	5	4	6	GS12584
4	2	4	3	5	3	3	5	7	3	3	3	5	4	6	GS12464
4	2	4	1	5	3	3	5	7	3	3	3	5	6	4	GS05108
4	2	4	1	5	3	3	5	7	3	3	3	5	6	4	GS05113
4	2	4	1	5	3	3	5	7	3	3	3	5	6	4	GS05133
4	2	4	1	5	3	3	5	7	3	3	3	5	6	4	GS05182
4	2	4	1	5	3	3	5	7	3	3	3	5	6	4	GS05184
4	2	4	1	5	3	3	5	7	3	3	3	5	6	4	GS05186
4	2	4	1	5	3	3	5	7	3	3	3	5	4	4	GS12174
4	2	4	1	5	3	3	5	7	3	3	3	5	4	5	GS12187
4	2	4	1	5	3	3	5	8	3	3	3	6	4	4	GS12447
4	2	4	1	5	3	3	5	7	3	3	3	6	4	4	GS12554
3	2	4	3	5	3	3	5	7	3	3	3	4	4	4	GS07019
3	2	4	3	5	3	3	5	7	3	3	3	4	4	4	GS07051
3	2	4	3	5	3	3	5	7	3	3	3	4	4	4	GS07054
3	2	4	3	5	3	3	5	7	3	3	3	1	4	4	GS07006
3	2	4	3	5	3	3	5	7	3	3	3	1	4	4	GS07046
3	2	4	3	5	3	3	5	7	3	3	3	1	4	4	GS12034
3	2	4	3	5	3	3	5	7	3	3	3	3	4	4	GS05127
3	2	4	3	5	3	3	5	7	3	3	3	3	4	4	GS05129
3	2	4	3	5	3	3	5	7	3	3	3	3	4	4	GS05150
3	2	4	3	5	3	3	5	7	3	3	3	3	4	4	GS05177
3	2	4	3	5	3	3	5	7	3	3	3	3	4	4	GS05185
3	2	4	3	5	3	3	5	7	3	3	3	5	4	4	GS12154
3	2	4	3	5	3	3	5	7	3	3	3	5	4	4	GS12240
3	2	4	3	5	3	3	5	7	3	3	3	5	4	4	GS12241
3	2	4	3	5	2	3	5	7	3	3	3	5	4	4	GS12349
3	2	4	3	5	3	3	5	7	3	3	3	5	4	5	GS07027
3	2	4	3	5	3	3	5	7	3	3	3	5	4	5	GS07058
3	2	4	3	5	3	3	5	7	3	3	3	5	4	5	GS07062
3	2	4	3	5	3	3	5	7	4	3	3	5	4	4	GS07028
3	2	4	3	5	3	2	5	7	3	3	3	3	4	4	GS05025
3	2	4	3	5	3	2	5	7	3	3	3	5	4	4	GS05057
3	2	4	3	5	3	2	5	7	3	3	3	6	4	4	GS05065
3	2	4	3	5	3	2	5	7	3	3	3	1	4	4	GS06084
3	2	4	3	5	3	3	5	7	3	2	3	6	4	4	GS12103
3	2	4	3	5	3	3	5	4	3	3	3	5	2	4	GS07048
3	2	4	3	5	3	3	5	8	3	3	3	5	2	4	GS12495
3	2	4	3	5	3	3	5	7	2	3	3	5	2	4	GS12357
3	2	4	3	5	3	3	5	7	2	3	3	5	2	4	GS12525
3	2	4	3	5	3	3	5	7	3	3	3	5	2	4	GS12110
3	2	4	3	5	3	3	5	7	3	3	3	5	2	4	GS12147
3	2	4	3	5	3	3	5	7	3	3	3	5	2	4	GS12545

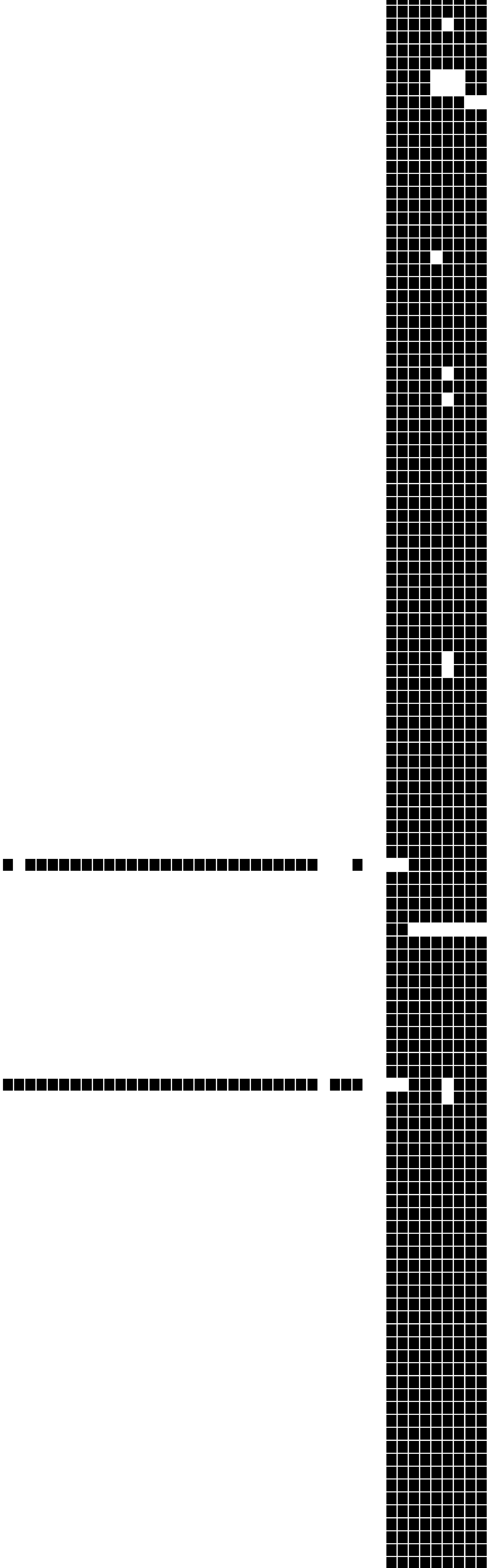

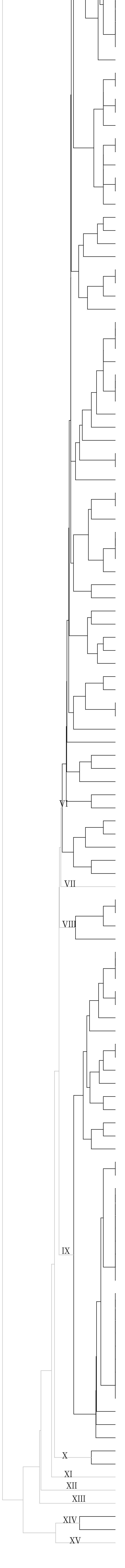

3	2	4	3	5	3	3	5	7	3	3	3	5	2	4	GS12147
3	2	4	3	5	3	3	5	7	3	3	3	5	2	4	GS12545
3	2	4	3	5	3	3	5	7	3	3	3	5	2	4	GS12549
3	2	4	3	5	3	3	5	7	3	3	3	5	2	4	GS12591
3	2	4	3	5	3	2	5	7	3	3	3	5	2	4	GS06041
2	2	4	3	5	3	2	5	5	3	3	3	4	4	4	GS06056
2	2	4	3	5	3	2	5	5	3	3	3	4	4	4	GS06057
2	2	4	3	5	3	2	5	7	3	3	3	4	4	4	GS06039
2	2	4	3	5	3	2	5	7	3	3	3	4	4	4	GS06073
2	2	4	3	5	3	2	5	9	3	3	3	4	4	4	GS06052
2	2	4	3	5	3	3	5	4	3	3	3	4	4	4	GS12246
2	2	4	3	5	3	3	5	4	3	3	3	4	4	4	GS12248
2	2	4	3	5	3	3	5	5	3	3	3	4	4	4	GS12593
2	2	4	3	5	3	3	5	7	3	3	3	4	4	4	GS12081
2	2	4	3	5	3	3	5	7	3	3	3	4	4	4	GS12391
2	2	4	3	5	3	3	5	6	3	3	3	4	4	4	GS07053
4	2	4	3	5	3	2	5	4	3	3	3	4	4	4	GS05039
4	2	4	3	4	3	2	5	4	3	3	3	4	4	4	GS12583
4	2	4	3	4	3	2	5	5	3	3	3	4	4	4	GS12596
4	2	4	3	6	3	4	5	5	3	3	3	4	4	4	GS12581
4	2	4	3	4	3	3	5	5	3	3	3	6	4	4	GS06099
4	2	4	3	4	3	3	5	5	3	3	3	6	4	4	GS07014
4	2	4	3	4	3	3	5	5	3	3	3	4	4	4	GS07049
4	2	4	3	5	3	3	6	5	3	3	3	6	4	4	GS12156
4	2	4	3	5	3	3	5	7	3	3	3	4	4	5	GS12061
4	2	4	3	5	3	3	5	7	3	3	3	4	4	5	GS12235
4	2	4	3	5	3	3	5	7	3	3	3	4	4	5	GS12527
4	2	4	3	4	3	3	5	7	3	3	3	4	4	5	GS12093
4	2	4	3	2	3	3	5	7	3	3	3	4	4	5	GS05086
4	2	4	3	2	3	3	5	7	3	3	3	4	4	5	GS12417
4	2	4	3	2	3	3	5	7	3	3	3	4	4	5	GS12430
4	2	4	3	2	3	3	5	7	3	3	3	4	4	4	GS07047
4	2	4	3	4	3	3	5	4	3	3	3	4	4	5	GS12029
4	2	3	3	5	3	3	5	5	3	3	3	4	4	5	GS12563
4	2	4	3	2	3	3	5	7	3	3	4	3	4	5	GS12083
4	2	4	3	2	3	3	5	7	3	3	4	3	4	5	GS12441
4	2	3	3	7	3	3	5	7	3	3	3	4	4	4	GS05098
4	2	4	3	5	1	3	5	7	3	3	3	4	2	4	GS12038
4	2	4	3	5	1	3	5	7	3	3	3	4	2	4	GS12411
4	2	4	3	5	1	3	4	7	3	3	3	4	4	4	GS12517
4	2	4	3	4	1	3	5	7	3	3	3	4	4	4	GS07038
4	2	4	3	4	1	3	5	7	3	3	3	4	4	4	GS12273
4	2	4	3	4	1	3	5	7	3	3	3	4	4	4	GS12485
4	2	4	3	4	1	3	5	6	3	3	3	4	4	4	GS12224
4	2	4	3	5	3	3	5	8	3	3	3	4	2	4	GS07034
4	2	4	3	5	3	3	5	7	3	3	2	4	2	4	GS12236
4	2	4	3	5	3	2	5	7	2	2	3	4	4	4	GS06003
4	2	4	3	5	3	2	3	7	3	2	3	4	4	4	GS06103
4	2	4	3	5	3	2	5	7	3	2	3	5	4	4	GS06061
4	2	4	3	5	3	2	5	7	3	2	3	3	4	4	GS06090
4	2	4	3	5	3	3	5	7	3	2	3	3	4	4	GS12004
4	2	4	3	5	3	1	5	7	3	3	3	6	4	4	GS06004
4	2	4	3	5	3	1	5	7	3	3	4	6	4	4	GS06012
4	2	4	3	3	3	4	5	7	3	3	3	6	4	4	GS12438
4	2	4	3	3	3	4	5	7	3	3	3	6	4	4	GS12548
4	2	4	3	3	2	3	5	7	3	3	2	6	4	4	GS12310
4	2	4	3	5	3	4	5	9	3	3	3	5	4	5	GS07042
4	2	4	3	3	3	3	5	6	3	3	3	5	4	3	GS07041
3	2	4	3	5	3	3	5	6	3	3	3	5	4	3	GS12561
3	2	4	3	5	2	3	5	7	3	3	3	5	4	3	GS12315
4	2	4	3	4	2	3	5	6	3	3	3	5	4	4	GS12472
4	2	4	3	7	2	3	5	6	3	3	3	6	4	4	GS12580
4	2	4	3	5	3	3	5	7	3	4	4	4	4	4	GS07036
4	2	4	3	5	3	3	5	7	3	3	4	4	4	4	GS12450
4	2	4	3	5	3	2	5	5	3	4	4	4	4	4	GS06020
4	2	4	3	5	3	2	5	7	3	3	4	4	4	5	GS05028
3	2	4	3	5	3	2	5	6	3	3	4	4	4	5	GS06072
4	3	4	3	5	3	2	5	7	3	3	3	4	3	4	GS06010
4	2	4	3	6	3	2	6	7	3	3	4	5	4	4	GS06068
4	2	4	3	6	3	2	6	7	3	3	4	5	4	4	GS06078
4	2	4	3	6	3	3	6	7	3	3	4	5	4	4	GS07001
4	2	4	2	6	3	2	5	7	3	3	3	5	4	4	GS06027
4	2	4	3	5	3	3	5	5	3	3	2	4	4	4	GS05130
4	2	4	3	5	3	3	5	5	3	3	2	4	4	4	GS05151
4	2	4	3	5	3	3	5	5	3	3	2	4	4	4	GS12254
4	2	4	3	5	3	2	5	5	3	3	2	4	4	4	GS05037
4	2	4	3	5	3	2	5	5	3	3	2	4	4	4	GS06009
4	2	4	3	5	1	3	5	5	3	3	2	4	4	4	GS12043
4	2	4	3	5	3	4	5	5	3	3	2	4	4	5	GS12373
4	2	4	3	5	3	4	5	6	3	3	2	4	4	4	GS06098
4	2	4	3	5	3	4	5	6	3	3	2	4	4	4	GS12106
4	2	4	3	5	3	3	5	6	3	3	2	4	4	4	GS06017
4	2	4	3	6	3	4	5	6	3	3	2	4	4	4	GS12456
4	2	4	3	5	1	2	5	6	3	3	2	4	4	4	GS05059
4	2	4	3	5	1	3	5	6	3	3	2	4	4	4	GS12308
4	2	4	3	4	3	4	5	5	3	3	2	4	4	4	GS07012
4	2	4	3	4	3	5	5	5	3	3	2	4	4	4	GS07015
4	2	4	3	4	3	3	5	7	3	3	2	4	4	4	GS05134
3	2	4	3	6	3	3	5	5	3	3	2	3	4	4	GS12244
3	2	4	3	6	3	3	5	5	3	3	2	3	4	4	GS12245
4	2	4	3	6	3	3	5	5	3	3	2	3	4	4	GS07003
4	2	4	3	6	3	3	5	5	3	3	2	3	4	4	GS07004
4	2	4	3	6	3	3	5	5	3	3	2	3	4	4	GS07016
4	2	4	3	6	3	3	5	5	3	3	2	3	4	4	GS07025
4	2	4	3	6	3	3	5	5	3	3	2	3	4	4	GS12022
4	2	4	3	6	3	3	5	5	3	3	2	3	4	4	GS12039
4	2	4	3	6	3	3	5	5	3	3	2	3	4	4	GS12443
4	2	4	3	6	3	3	5	5	3	3	2	3	4	4	GS12483
4	2	4	3	6	3	2	5	5	3	3	2	3	4	4	GS05051
4	2	4	3	6	3	2	5	5	3	3	2	3	4	4	GS05060
4	2	4	3	6	3	2	5	5	3	3	2	3	4	4	GS06001
4	2	4	3	6	3	2	5	5	3	3	2	3	4	4	GS06002
4	2	4	3	6	3	2	5	5	3	3	2	3	4	4	GS06014
4	2	4	3	6	3	2	5	5	3	3	2	3	4	4	GS06044
4	2	4	3	6	3	2	5	5	3	3	2	3	4	4	GS06081
4	2	4	3	6	3	2	5	5	3	3	2	3	4	4	GS06082
4	2	4	3	6	3	2	5	5	3	3	2	3	4	4	GS06101
4	2	4	1	6	3	3	5	5	3	3	2	3	4	4	GS12019
4	2	4	3	6	3	3	5	5	3	3	2	3	4	5	GS12098
4	2	4	3	6	3	3	5	4	3	3	2	3	4	4	GS12550
3	2	3	3	5	3	3	6	7	3	3	4	1	4	4	GS12053
3	2	4	3	5	3	3	5	7	3	3	4	1	4	4	GS12513
3	2	4	3	5	3	2	5	3	3	3	3	2	4	6	GS06079
4	2	4	3	4	3	3	6	5	4	3	3	4	2	4	GS12152
4	2	2	3	5	6	4	5	4	3	3	3	4	2	4	GS12173
3	2	4	3	3	4	1	5	1	3	2	2	3	4	4	GS12008
5	2	4	3	3	3	2	5	1	3	2	2	3	4	4	GS12508
3	2	4	3	2	5	1	5	1	3	2	2	5	4	5	GS12471

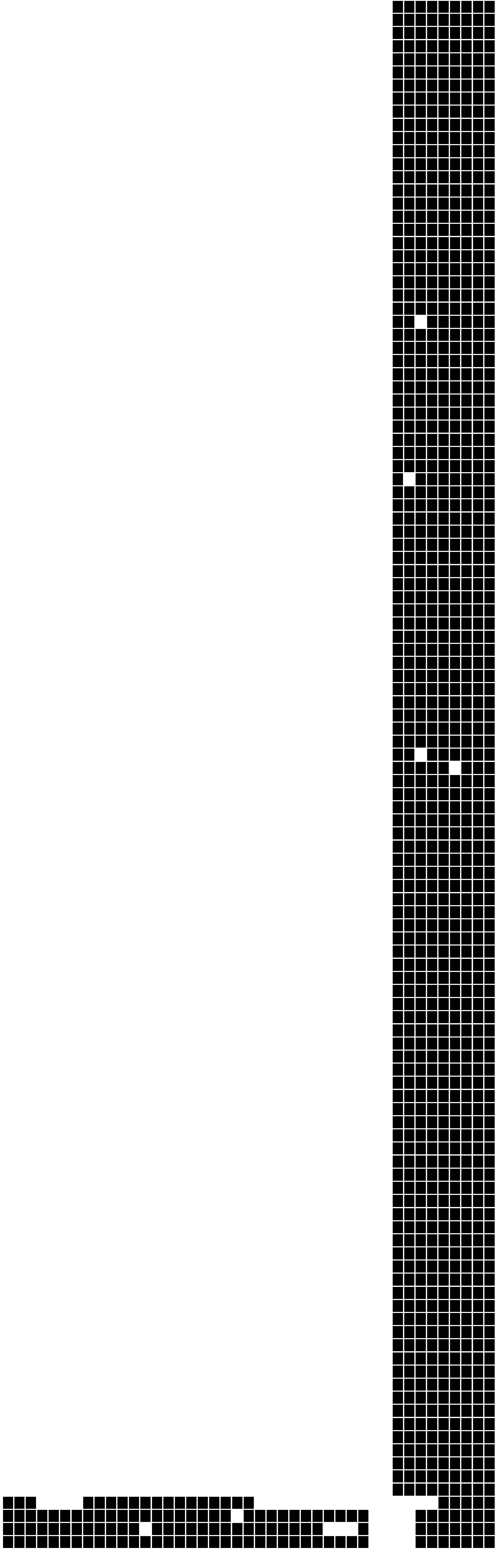

Supplement: Figure S1 — Genotyping of 467 M. tuberculosis isolates with VNTR15-China and Spoligotyping. The clustering was based on the analysis performed using BioNumerics 6.5 to compare these two genotyping methods. From left to right: 1) UPGMA dendrogram generated by VNTR15-China 2) the repeat number in each VNTR-locus 3) spoligotyping patterns 4) strain No. (PDF) [file pone.0099357.s001.pdf]
